# Evaluating large language models as tools for public health education on scoliosis

**DOI:** 10.3389/fpubh.2026.1873062

**Published:** 2026-07-17

**Authors:** Yi chen Wang, Xuanyan Liu, Mengjie Chen, Fangchun Jin, Xiaohan Wu, Yi Luo

**Affiliations:** 1 Department of Orthopedics, School of Medicine, Shanghai Children's Hospital, Shanghai Jiao Tong University, Shanghai, China; 2 Department of Administration, School of Medicine, Shanghai Children’s Hospital, Shanghai Jiao Tong University, Shanghai, China; 3 Department of Pediatric Orthopedics, Xin Hua Hospital Affiliated to Shanghai Jiao Tong University School of Medicine, Shanghai, China; 4 Department of Pediatric Surgery, Zhaotong First People’s Hospital, Zhaotong, Yunnan, China

**Keywords:** artificial intelligence, frequently asked questions, large language models, scoliosis, society on scoliosis orthopedic and rehabilitation treatment

## Abstract

**Purpose:**

This study was aimed to compare the efficacy of three most popular large language models (LLMs)—Claude Opus 4.6, ChatGPT Thinking 5.4 and DeepSeek v3.2 in answering frequently asked questions (FAQs) about scoliosis.

**Methods:**

20 scoliosis related questions (four categories, five questions in each category) were submitted to each LLM. A panel of 9 experts (two spine surgeons, two pediatric orthopedic surgeons and five physical therapists, all blinded to the LLMs and responses) rated independently each response generated by LLMs on a 6 points Likert scale (1 as strongly disagree to 6 as strongly agree). 540 total ratings were collected. Intergroup comparisons were conducted by Kruskal Wallis test and Mann Whitney U pairwise tests. Paired question level analysis was achieved by Friedman test and Wilcoxon signed rank comparisons.

**Results:**

Claude’s score was 5.53 ± 0.76 much higher than both ChatGPT (4.84 ± 0.86, *p* < 0.001) and DeepSeek (4.86 ± 0.84, *p* < 0.001), but no difference was found between ChatGPT and DeepSeek (*p* = 0.749). Claude performed on top for 19 of 20 questions (95%) and was favored by 7 of 9 reviewers. Consistency of Claude was also highest [CV = 13.8% vs. 17.9% (ChatGPT) and 17.2% (DeepSeek)].

**Conclusion:**

Although all three LLMs achieved favorable overall ratings (>4.8/6), Claude performed significantly better than ChatGPT and DeepSeek for scoliosis FAQs taking into account its higher accuracy and consistency. Within the scope of the present evaluation, Claude demonstrated the strongest overall performance among the three LLMs tested.

## 1 Introduction

Adolescent idiopathic scoliosis (AIS) is a three-dimensional spine deformity. The incidence of AIS is around 1 to 3% according to different reports (1–4). With the rapid development of large language models (LLMs), there is a trend that patients and caregivers are choosing to inquire AI chatbots for medical guidance before and after their vists to the clinic due to ease of accessibility (5–8), because it’s too demanding for the patients and caregivers to check classic guidelines about screening, brace and exercise programs (9–11).

Recent studies have evaluated several individual LLM’s performance in handling scoliosis related FAQs. Giray et al. (12) found that ChatGPT 3.5 was correct in 53.1% of AIS questions with strongest performance for quality-of-life questions (86.7%) but poor accuracy on treatment recommendations (26.3%). Suresh et al. (13) compared ChatGPT, Gemini and Copilot, confirming ChatGPT to be significantly superior(The author did not mention the versions of LLMs). In another study (14), ChatGPT 4.0 achieved content validity in 78% of responses with high clarity ratings on conservative scoliosis treatment, but content validity was low when addressing questions in exercise and sport recommendations (15–19).

Both Claude Opus 4.6 and DeepSeek V3.2 performed impressively well on a wide range of benchmarks. Nevertheless, neither Claude Opus 4.6 (Anthropic) nor DeepSeek v3.2 has been tested in the scoliosis patient education space up till now. We set out to run the first direct comparison of Claude Opus 4.6, ChatGPT Thinking 5.4, and DeepSeek v3.2 on scoliosis related FAQs.

## 2 Methods

### 2.1 Study design

This study was conducted between March and April 2026 and was designed as an observational and prospective study. Ethical approval was not required due to the nature of the study since no patient data were collected and all participants were professionals voluntarily. The study was conducted entirely in English.

### 2.2 Question selection

20 scoliosis related questions (q1–q20) were compiled from published sources including Scoliosis Research Society (SRS), American Academy of Orthopedic Surgeons (AAOS) and Society on Scoliosis Orthopedic and Rehabilitation Treatment (SOSORT) consensus documents (1). The complete list of the 20 evaluated scoliosis-related questions is provided in Table 1.

**Table 1:** 20 scoliosis-related FAQs; Group 1: Q1-Q5, basic information; Group 2: Q6-Q10, diagnoses; Group 3: Q11-Q15, treatment; Group 4: Q16-Q20, living with scoliosis.

No	Question
1	What is scoliosis?
2	What are the causes of scoliosis?
3	Does bad posture cause scoliosis?
4	Does scoliosis run in family?
5	When does scoliosis usually appear?
6	What are the signs of scoliosis?
7	How is scoliosis diagnosed?
8	What is Cobb angle?
9	Does scoliosis hurt?
10	Can scoliosis affect organs like lung and heart?
11	Should scoliosis be treated?
12	What’s the purpose of a brace?
13	Is physical therapy helpful to my child’s scoliosis?
14	When is surgery needed for scoliosis?
15	What is posterior spinal fusion (PSF)?
16	Can I play sports and exercise with scoliosis?
17	Will scoliosis affect my pregnancy?
18	Can scoliosis progress in adulthood?
19	Is it possible to live a normal life with scoliosis?
20	Are there activities to avoid with scoliosis?

Group 1 (q1–q5) covered basic information (what scoliosis is, its causes, posture and genetic factors and typical age of onset); Group 2 (q6–q10) covered symptoms and diagnosis (warning signs, diagnostic methods, Cobb angle, pain and organ involvement); Group 3 (q11–q15) covered treatment and management (need for treatment, bracing purpose, role of physical therapy, surgical indications and spinal fusion) and Group 4 (q16–q20) covered living with Scoliosis (sports participation, pregnancy, adult progression, daily living and exercise precautions). Questions were derived from established educational resources and guidelines to ensure standardization and coverage of commonly recognized scoliosis topics.

### 2.3 AI platforms

Three LLMs were evaluated: Claude Opus 4.6, developed by Anthropic; ChatGPT Thinking 5.4, developed by OpenAI; and DeepSeek v3.2 by Hangzhou DeepSeek Artificial Intelligence Basic Technology Research. Each question was queried once per platform, reflecting a standardized real-world scenario in which a patient or caregiver typically submits a single question and receives a single response. We did not use any system prompts, role instructions or other constraints. Responses were recorded exactly as given without editing (5, 6).

### 2.4 Expert evaluation

9 expert reviewers (Reviewer 1–9), blinded to the responses given by LLMs, independently scored every answer on a 6-point Likert scale (1 = strongly disagree, 2 = disagree, 3 = slight disagree, 4 = slight agree, 5 = agree and 6 = strongly agree). The panel included two spine surgeons, two pediatric orthopedic surgeons and five physical therapists, all experienced over 10 years in scoliosis care. Each reviewer rated all 60 responses (20 questions × 3 LLMs). A total of 540 ratings were collected finally. We also randomized the order of responses given to the experts to avoid ordering effects.

### 2.5 Statistical analysis

Statistical analyses were performed by SPSS software (v 26.0, IBM, Armonk, NY). Intergroup comparisons were conducted by Kruskal Wallis test and Mann Whitney U pairwise tests. Paired question level analysis was achieved by Friedman test and Wilcoxon signed rank comparisons. Consistency was assessed by coefficient of variation (CV, calculated as SD/mean × 100%). Inter-rater reliability was assessed by multiple metrics: Fleiss’ kappa, intraclass correlation coefficient (ICC; two-way random effects) for both single measure ICC (2,1) and average measure ICC (2, k), Kendall’s coefficient of concordance, Krippendorff’s alpha and percentage agreement (both exact and within ±1 point). Significance level was set at *p* < 0.05 (see Table 2).

**Table 2:** Inter-rater reliability metrics across three LLMs and overall (9 reviewers).

Metric	Claude Opus 4.6	ChatGPT Thinking 5.4	DeepSeek v3.2	Overall	Interpretation
Fleiss’ kappa	0.022	0.009	−0.016	**0.107**	Slight
ICC(2,1)	0.047	0.037	−0.000	**0.14**	Poor (single)
ICC(2,k)	0.308	0.255	−0.000	**0.595**	Moderate (avg)
Kendall’s W	0.086	0.114	0.081	0.226***	Fair concordance
Krippendorff’s α	0.028	0.015	−0.010	**0.109**	Low reliability
Exact agreement (%)	**50.80%**	34.40%	37.90%	—	—
Agreement ±1 (%)	**86.20%**	79.60%	83.80%	—	—

ICC, intraclass correlation coefficient; ***p<0.001. ICC(2,1) = single-measure two-way random; ICC(2,k) = average-measure two-way random. Bold values indicate overall (combined) metrics.

## 3 Results

### 3.1 Overall performance

540 ratings were evaluated in total. Claude achieved the highest mean score of 5.53 ± 0.76, compared with ChatGPT (4.84 ± 0.86) and DeepSeek (4.86 ± 0.84). Kruskal Wallis test showed a significant difference among the three models (*p* < 0.001).

**Figure 1:**
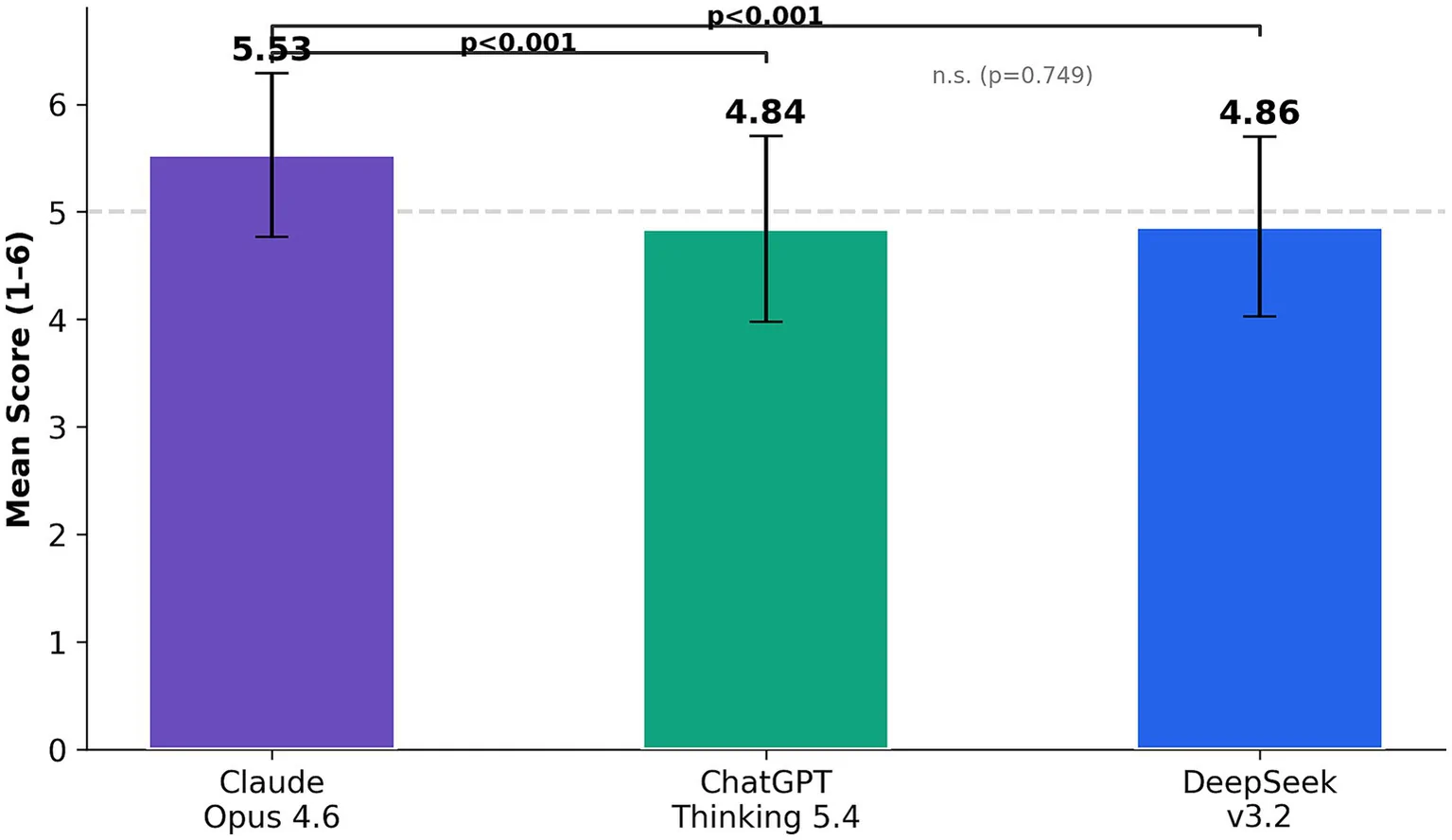
Overall mean scores for each LLM. Claude Opus 4.6 significantly outperformed both ChatGPT Thinking 5.4 and DeepSeek v3.2 (*p* < 0.001). No significant difference between ChatGPT Thinking 5.4 and DeepSeek v3.2 (*p* = 0.749).

In pairwise comparisons, Claude significantly outperformed both ChatGPT (*p* < 0.001) and DeepSeek (*p* < 0.001). There was no significant difference between ChatGPT and DeepSeek (*p* = 0.749) (Figure 1).

### 3.2 Score distribution

**Figure 2:**
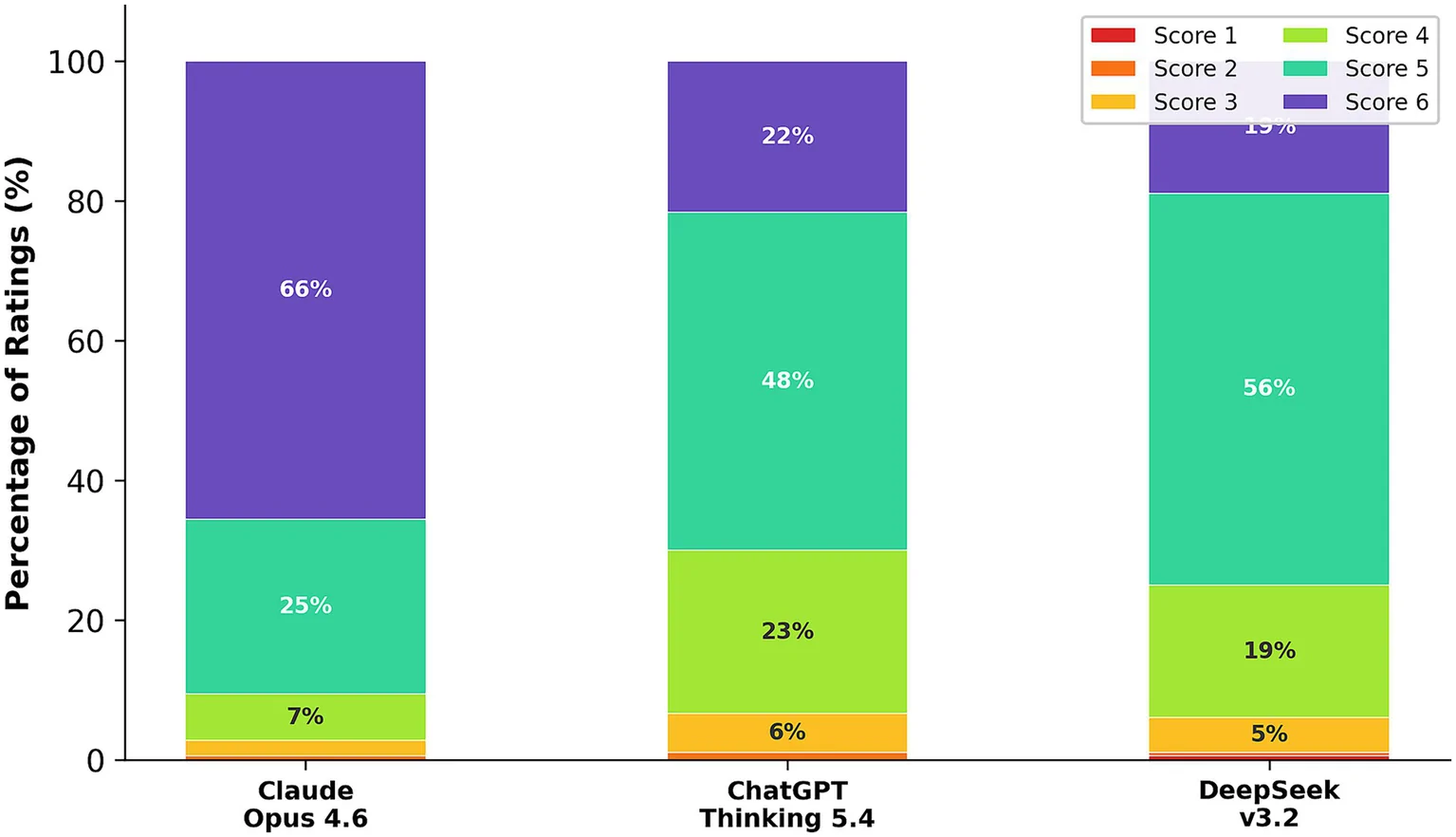
Score distribution by LLM. Claude Opus 4.6 received the maximum score (6) in 65.6% of ratings, approximately three times the rate of either competitor.

Claude Opus 4.6 showed a pronounced ceiling effect as 65.6% of the ratings get the maximum score of 6 and 90.6% were at 5 or above. By comparison, ChatGPT reached top score in just 21.7% cases with most ratings at 5 (48.3%). DeepSeek looked similar—18.9% at score 6 and most at 5 (56.1%). Scores of 3 or below were uncommon: 2.8% for Claude, 6.7% for ChatGPT and 6.1% for DeepSeek (Figure 2).

### 3.3 Question level analysis

**Figure 3:**
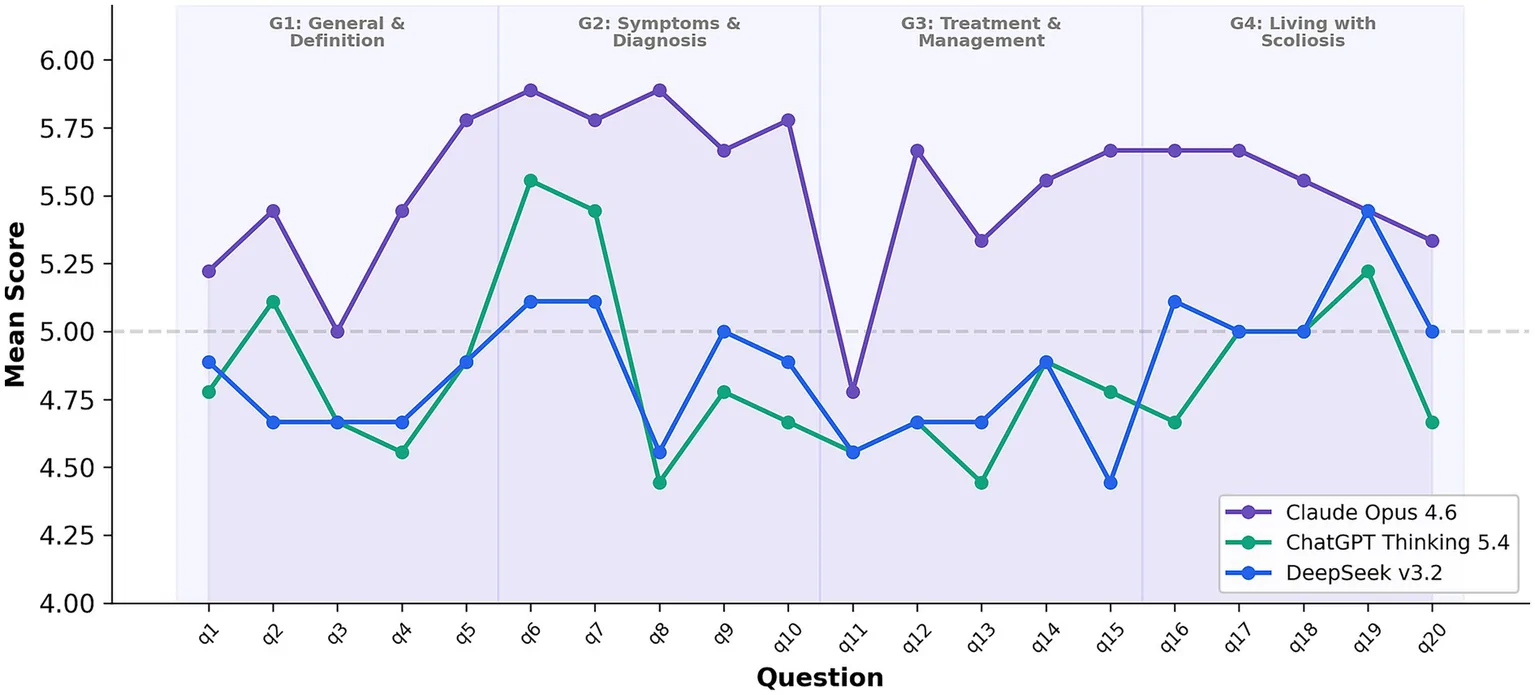
Mean scores across all 20 scoliosis questions. Claude Opus 4.6 (purple) consistently scored above both ChatGPT Thinking 5.4 (green) and DeepSeek v3.2 (blue).

Claude achieved the highest score on 19 of 20 questions (95%), with one tie on q19 (Claude and DeepSeek both at 5.44). Neither ChatGPT nor DeepSeek v3.2 came out on top for any question on its own. The Friedman test confirmed a significant effect of model type across questions (*χ*^2^ = 32.53, *p* < 0.001). Wilcoxon signed rank tests showed Claude significantly outperformed both ChatGPT (*W* = 0, *p* < 0.001) and DeepSeek (W = 0, *p* < 0.001), while ChatGPT and DeepSeek did not differ significantly (*W* = 43, *p* = 0.861) (Figure 3).

Claude Opus 4.6’s highest score went to q6 “What are the warning signs of scoliosis?” and q8 “What is a Cobb angle?” (mean 5.89 each), while its lowest was q11 “Does scoliosis need to be treated?” (mean 4.78), still higher than both competitors. ChatGPT’s best performance was on q6 (5.56) and its weakest on q8 “What is a Cobb angle?,” q11 “Does scoliosis need to be treated?” and q13 “Will physical therapy or exercise help straighten my spine?” (all 4.44). DeepSeek best was q19 “Is it possible to live a normal life with scoliosis?” (5.44) and worst was q15 “What is spinal fusion surgery?” (4.44).

### 3.4 Reviewer level analysis

**Figure 4:**
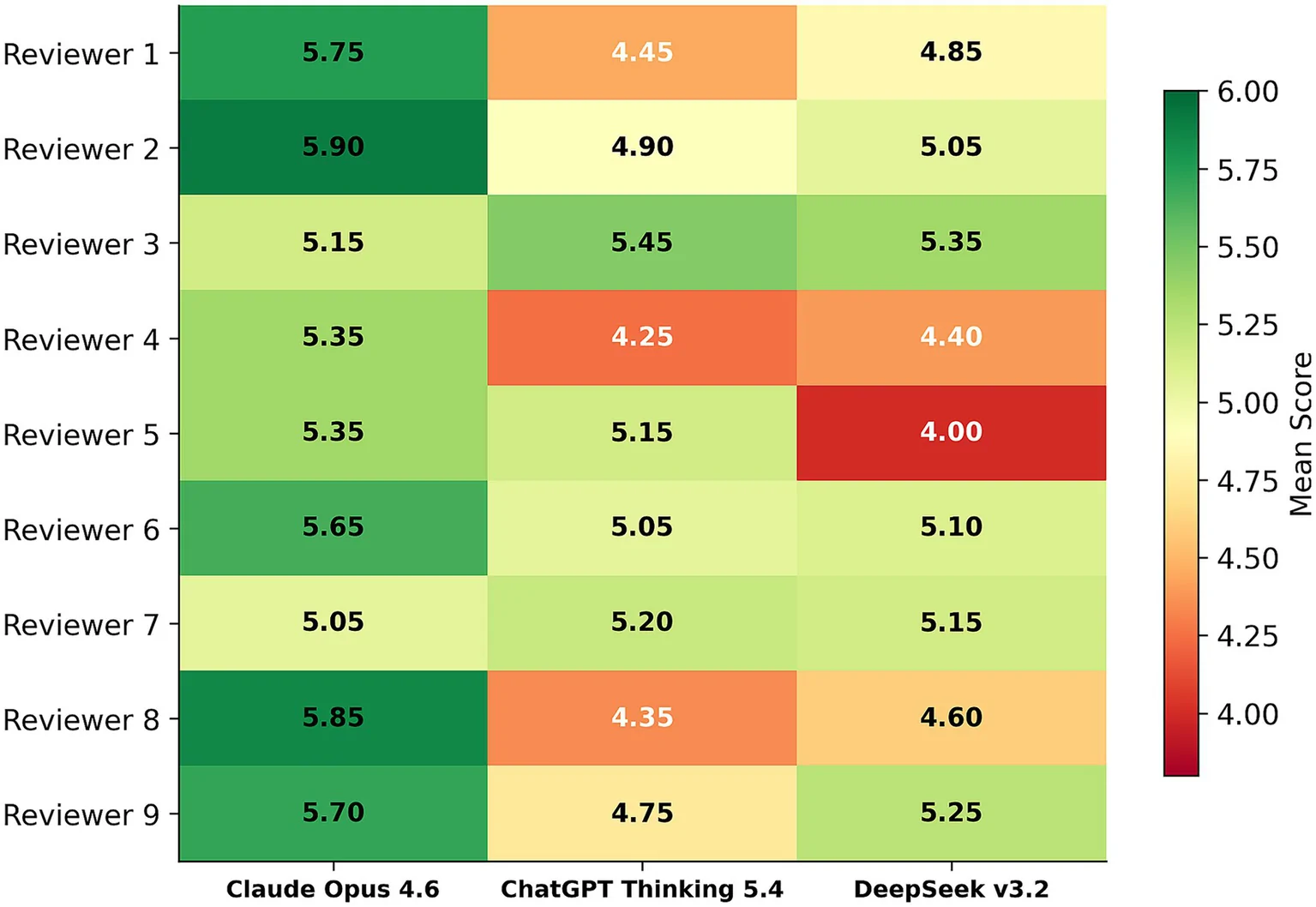
Heatmap of mean scores across 9 reviewers and 3 LLMs. Warmer colors (green) indicate higher scores. Claude Opus 4.6 received the highest scores from 7 of 9 reviewers.

Claude was ranked first by 7 of 9 reviewers (77.8%). Two reviewers ranked ChatGPT first, with Claude still within 0.40 points in both cases. No reviewer ranked DeepSeek first. The heatmap shows that Claude’s advantage was particularly pronounced for Reviewers 2, 8, 1 and 9 (Figure 4).

### 3.5 Question group analysis

**Figure 5:**
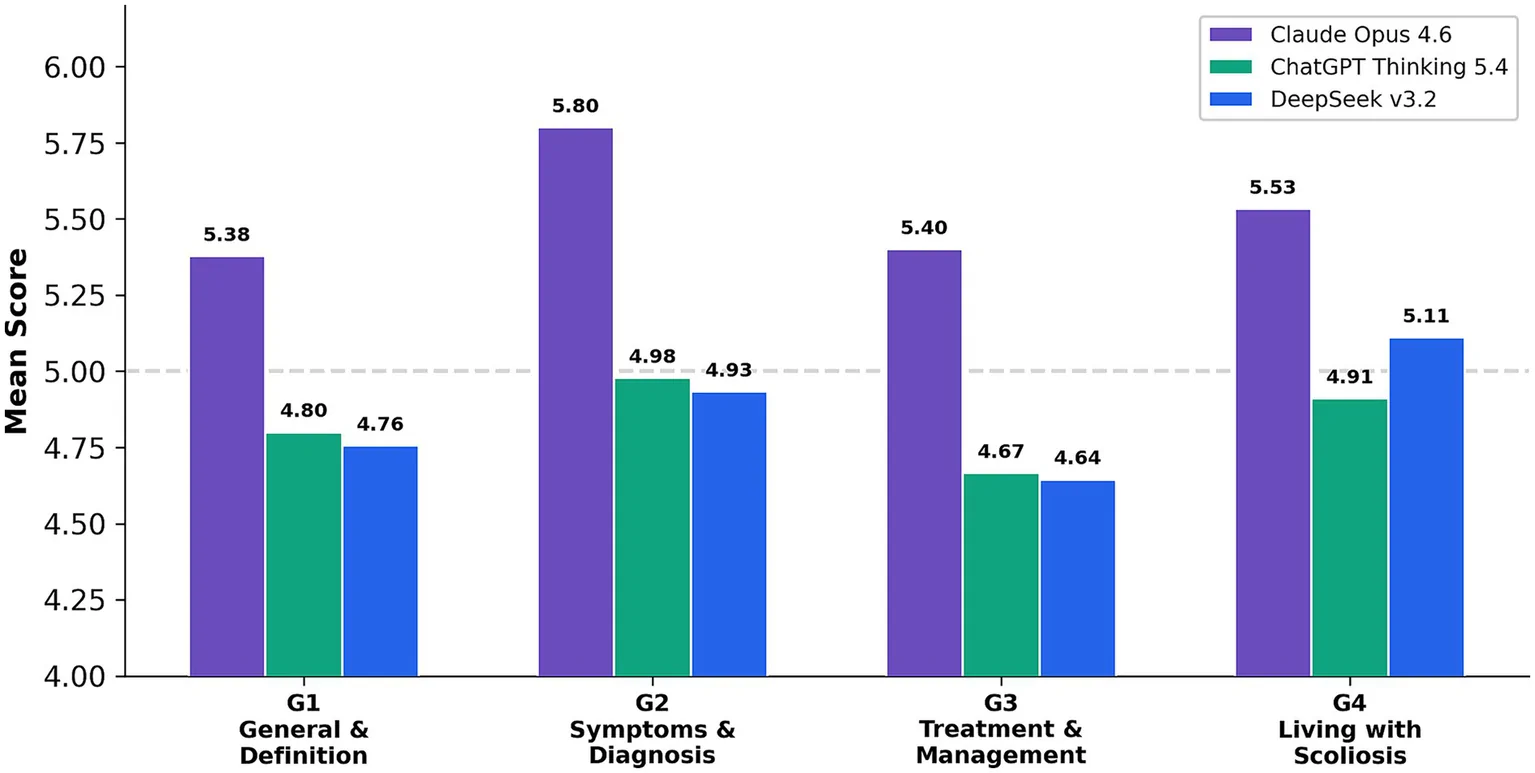
Mean scores by question group. G1 = General & Definition, G2 = Symptoms & Diagnosis, G3 = Treatment & Management, G4 = Living with Scoliosis. Claude Opus 4.6 maintained superiority across all four groups. All platforms exhibited a peak in G2 (Symptoms & Diagnosis) and dip in G3 (Treatment & Management).

We also broke down performance by the four question groups. Claude maintained the highest across all four groups: G1 General & Definition = 5.38, G2 Symptoms & Diagnosis = 5.80, G3 Treatment & Management = 5.40 and G4 Living with Scoliosis = 5.53 (Figure 5). All three LLMs showed a consistent sawtooth pattern, peaking in G2 and dipping in G3. DeepSeek showed the most pronounced recovery from G3 to G4 (4.64 to 5.11). ChatGPT and DeepSeek performed closely throughout all groups, diverging < 0.07 points.

### 3.6 Consistency and multi-dimensional profile

**Figure 6:**
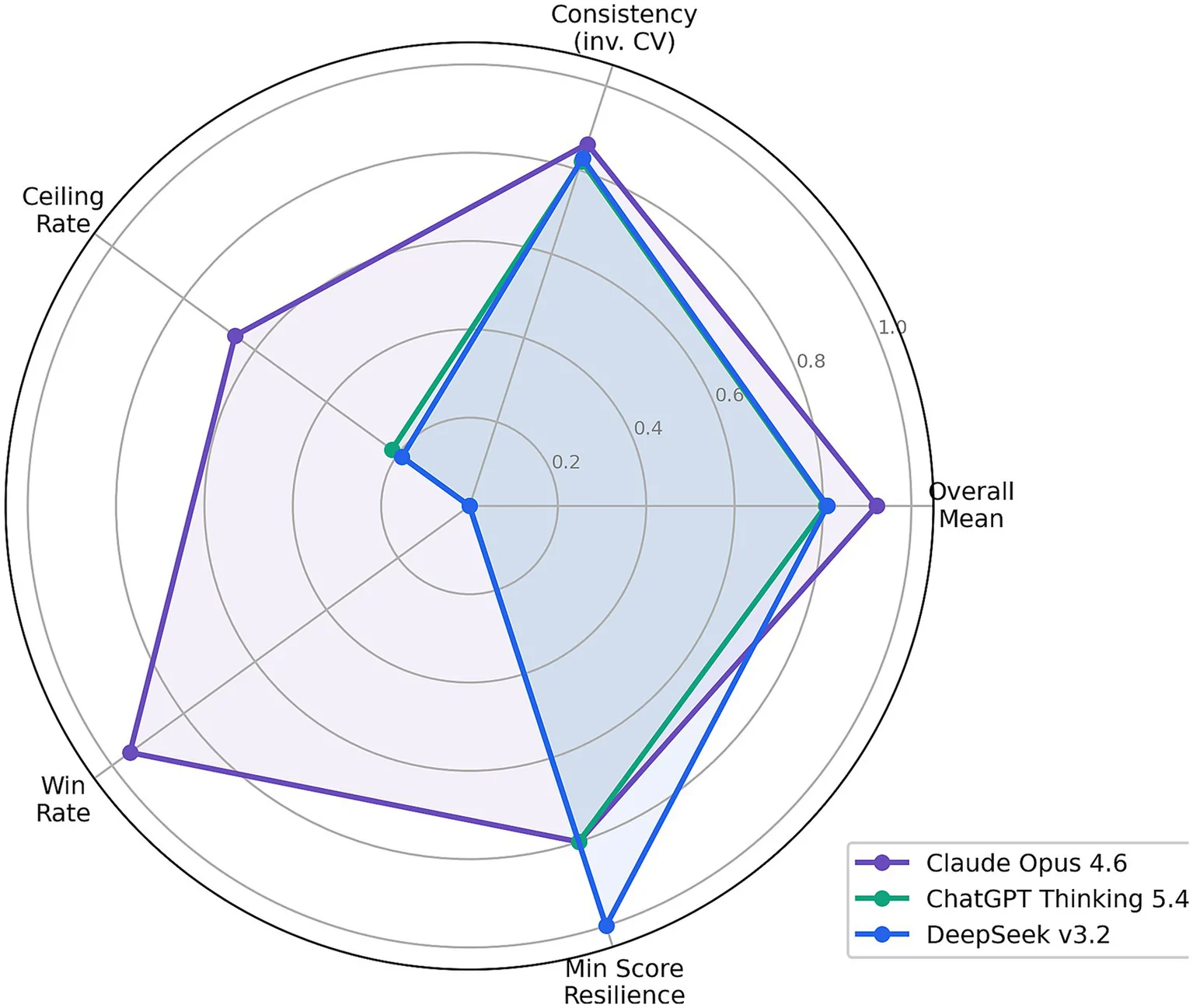
Multi-dimensional radar chart. Claude Opus 4.6 (purple) outperforms ChatGPT Thinking 5.4 (green) and DeepSeek v3.2 (blue) across all five normalized performance dimensions.

Claude was also the most consistent (CV = 13.8%) versus for ChatGPT (17.9%) and for DeepSeek (17.2%). A radar chart (Figure 6) pulling together five normalized dimensions—overall mean, consistency, ceiling rate, win rate and minimum-score resilience—shows Claude leading on every aspect.

### 3.7 Inter-rater reliability

Inter-rater reliability was assessed using multiple metrics. Across all three LLMs, agreement on exact scores was slight (Fleiss’ *κ* = 0.107), but rank-order agreement was significant (Kendall’s W = 0.226, *p* < 0.001), indicating reviewers generally agreed on relative quality despite differing scores. Reliability was moderate when ratings were averaged (ICC (2, k) = 0.595) but low for individual raters (ICC (2, 1) = 0.140).

Within individual models, reliability was low across all measures. Fleiss’ kappa ranged from −0.016 to 0.022 and Kendall’s W was non-significant for each model, suggesting near-chance agreement. The higher agreement when models were pooled (*W* = 0.226 vs. nonsignificant individually) indicates that differences were driven more by variation between models than within them.

Exact agreement rates were 50.8% for Claude, 37.9% for DeepSeek and 34.4% for ChatGPT. Allowing a ± 1-point difference increased agreement substantially (86.2, 83.8 and 79.6%, respectively). Higher exact agreement for Claude likely reflects score clustering at the top end.

## 4 Discussion

To our knowledge, this is the first study to directly compare Claude Opus 4.6, ChatGPT Thinking 5.4, and DeepSeek v3.2 (three most popular LLMs in the last year) in the context of scoliosis patient education. The results were clear: Claude Opus 4.6 significantly outperforms the other two.

Claude’s advantage held up across every angle we examined. It ranked first on 95% of questions, was top-rated among LLMs for 78% of reviewers and maintained superiority across all four question groups. Its mean score advantage was 0.69 points, which can be translated to a meaningful difference on a 6-point scale. Almost two thirds (65.6%) of Claude’s ratings reached the maximum score of 6. That was roughly three times the rate of either competitor, suggesting that experts often found its responses excellent with little to add.

The lack of significant difference between ChatGPT and DeepSeek (*p* = 0.749) is worth highlighting. Earlier work had consistently placed ChatGPT at the front of the pack for medical question-answering. Suresh et al. found ChatGPT significantly outperforming Google Gemini and Microsoft Copilot (10). The fact that DeepSeek matched ChatGPT here suggests that newer open-source models have caught up with established commercial ones in this particular domain, despite the fact that both still trail Claude by a meaningful margin.

Claude’s lower coefficient of variation (13.8% vs. 17.5% for competitors) matters for real world use. Speaking of a patient education tool, consistency may be just as important as average accuracy. An LLM that sometimes shines but occasionally gives a weak answer could be more problematic than one that reliably delivers solid responses. Claude’s high average score combined with lower variability suggests that it may currently provide more consistent responses than the other models evaluated in this study (15, 20).

The inter-rater reliability findings also offer a useful methodological lesson. Intra-LLM agreement was uniformly low across all metrics (Fleiss’ *κ* ranging from −0.016 to 0.022), which was consistent with Negrini et al. (14) who also found only slight agreement (Fleiss’ κ = 0.10) among their 29-expert panel. However, reliability improved a lot once the three LLMs were evaluated together: Kendall’s W jumped to a significant 0.226 (*p* < 0.001), and the ICC (2, k) rose to a moderate 0.595. In practical terms, the reviewers generally agreed on which model gave the better answers—they just did not see eye-to-eye on the exact scores to assign. The signal of Claude’s edge came through clearly despite the noise in individual ratings. The low within-LLM agreement probably comes down to the inherent subjectivity of grading medical content on a fine-grained scale, compounded by the multidisciplinary composition of the panel (surgeons vs. physical therapists may weight different aspects of a response). This underscores why it is important to use several complementary reliability metrics and a reasonably large reviewer panel in studies like this.

The pattern of all three LLMs peaking in G2 and dipping in G3 has a straightforward clinical explanation. G2 contained factual, well-defined topics such as warning signs, diagnostic methods, Cobb angle, pain and organ involvement. These are questions with well established, consensus-backed answers that are likely well represented in training data. In contrast, G3 addressed more nuanced clinical questions including treatment necessity, bracing purpose, the role of physical therapy, surgical indications and spinal fusion. Unfortunately, these are topics where guidelines differ and expert opinion is less settled (1, 9–11). This finding is consistent with Giray et al. (12), who identified treatment and follow-up as the weakest domain for ChatGPT-3.5 (26.3% in accuracy), and Negrini et al. (14), who found low inter-rater agreement among experts on treatment related questions.

### 4.1 Comparison with prior literature

These findings add to the existing literature in a few important respects. First, it is the first evaluation of Claude for scoliosis patient education and it turned out to be the strongest performer of any LLM tested so far. Second, including DeepSeek shows that open-source models can now match ChatGPT in medical accuracy, which challenges the assumption that commercial LLMs are inherently better. Third, our nine-reviewer multidisciplinary panel—spine surgeons, pediatric orthopedic surgeons and physical therapists, well beyond the 2–4 reviewers typical of earlier studies (12, 13)—offers broader and more clinically representative perspectives.

Our findings are also consistent with observations from other spine-related patient education studies. Tabanli and Demirkiran compared ChatGPT 3.5 and ChatGPT 4.0 in low back pain patient education and reported that LLMs were effective in delivering factual information but showed limitations when addressing psychosocial concerns and individualized patient contexts. Similarly, our results suggest that current LLMs may serve as useful educational adjuncts, although professional clinical guidance remains essential for personalized decision-making and complex treatment discussions (21).

Our findings are also consistent with emerging evidence from other musculoskeletal specialties. Kaya et al. compared GPT-4o and GPT-5 in sports injury care using expert assessment and similarly demonstrated that contemporary LLMs can provide generally high-quality medical information while still exhibiting variability across clinical scenarios. Together with our findings in scoliosis patient education, these studies suggest that LLM evaluation should be conducted within disease-specific contexts rather than assuming uniform performance across healthcare domains (22).

### 4.2 Limitations

We have to admit that this study has several limitations worth noting. First, each question was queried only once. Because LLMs generate probabilistic outputs, repeated prompting may produce different responses. Consequently, the present study did not assess intra-model response variability or reproducibility. Future investigations should evaluate response stability through repeated querying under standardized conditions. Second, although our multidisciplinary panel included both surgical and rehabilitation specialists and was broader than panels used in several previous scoliosis-related LLM studies, physical therapists constituted a slight majority of reviewers (5 of 9). This imbalance may have influenced scoring patterns, particularly for questions involving conservative treatment, exercise-based interventions, and rehabilitation strategies. Future studies with a more balanced distribution of reviewer specialties may further improve generalizability. Third, we used a single composite quality score rather than evaluating separate domains such as factual accuracy, comprehensiveness, readability, and clinical usefulness. Although this approach simplified the assessment process and reduced reviewer burden, because each reviewer assessed 60 responses, it may have masked differences in specific attributes of response quality. Future studies should incorporate multidimensional evaluation frameworks to better characterize the strengths and weaknesses of different LLMs. Fourth, response length and readability were not formally evaluated. Since patient education materials should be understandable to individuals with diverse educational backgrounds, future studies should incorporate objective readability metrics, response-length analyses, and linguistic accessibility assessments alongside expert ratings (13). Finally, the evaluated questions were derived from published educational resources and professional guidelines rather than directly collected from patients or caregivers. Although this approach improved standardization, it may not fully reflect the diversity, wording, and practical concerns encountered in real-world clinical settings. Future studies incorporating patient-generated questions may provide a more realistic assessment of LLM performance.5. Conclusions.

Across every analysis we ran, Claude Opus 4.6 was significantly better than both ChatGPT Thinking 5.4 and DeepSeek v3.2 at answering scoliosis-related FAQs. It ranked first on 95% of questions, earned top score about three times as often as either competitor, was the most consistent and was the preferred model for 78% of reviewers. ChatGPT Thinking 5.4 and DeepSeek v3.2 performed about equally (*p* = 0.749), which suggests that the medical AI landscape has moved past the era of clear ChatGPT dominance.

All three models performed adequately overall (means above 4.8 on a 6-point scale), among the three LLMs evaluated, Claude Opus 4.6 demonstrated the strongest overall performance for scoliosis-related patient education questions. However, ongoing model updates may alter comparative performance over time. Nevertheless, LLM-generated medical information should still be treated as a supplement to, not a substitute for, professional clinical advice, especially when it comes to treatment decisions, where AI tools have well-documented shortcomings.

## Data Availability

The datasets analyzed in this study are available from the corresponding author upon reasonable request.
