# Correction: Evaluating large language models as tools for public health education on scoliosis

**DOI:** 10.3389/fpubh.2026.1943276

**Published:** 2026-08-07

**Authors:** Yi chen Wang, Xuanyan Liu, Mengjie Chen, Fangchun Jin, Xiaohan Wu, Yi Luo

**Affiliations:** 1 Department of Orthopedics, School of Medicine, Shanghai Children's Hospital, Shanghai Jiao Tong University, Shanghai, China; 2 Department of Administration, School of Medicine, Shanghai Children's Hospital, Shanghai Jiao Tong University, Shanghai, China; 3 Department of Pediatric Orthopedics, Xin Hua Hospital Affiliated to Shanghai Jiao Tong University School of Medicine, Shanghai, China; 4 Department of Pediatric Surgery, Zhaotong First People's Hospital, Zhaotong, Yunnan, China

**Keywords:** artificial intelligence, frequently asked questions, large language models, scoliosis, society on scoliosis orthopedic and rehabilitation treatment

Authors Yi chen Wang and Xuanyan Liu were erroneously omitted as equal contributing authors.

The original version of this article has been updated.

